# Response to the letter: Transoral Endoscopic Thyroidectomy Vestibular Approach (TOETVA): Pioneers's Point of View

**DOI:** 10.20945/2359-3997000000425

**Published:** 2021-11-24

**Authors:** Alfio Tincani, Carlos Lehn, Cláudio Cernea, Emilson Queiroz, Fernando Dias, Fernando Walder, Flávio Hojaij, Francisco Monteiro, Jacob Kligerman, José Podesta, Lenine Brandão, Luiz Eduardo Barbalho de Mello, Luiz Medina, Márcio Abrahao, Marcos Tavares, Mauro Barbosa, Onivaldo Cervantes, Paula Demétrio, Ricardo Curioso, Roberto Lima, Sérgio Arap, Sylvio Vasconcellos

**Affiliations:** 1 Universidade Estadual de Campinas Campinas SP Brasil Universidade Estadual de Campinas, Campinas, SP, Brasil; 2 Hospital do Servidor Público Estadual de São Paulo São Paulo SP Brasil Hospital do Servidor Público Estadual de São Paulo, São Paulo, SP, Brasil; 3 Universidade de São Paulo Faculdade de Medicina Departamento de Cirurgia São Paulo SP Brasil Departamento de Cirurgia da Faculdade de Medicina da Universidade de São Paulo, São Paulo, SP, Brasil; 4 Instituto Nacional de Câncer Rio de Janeiro RJ Brasil Instituto Nacional de Câncer, Rio de Janeiro, RJ, Brasil; 5 Universidade Federal de São Paulo Escola Paulista de Medicina São Paulo SP Brasil Universidade Federal de São Paulo, Escola Paulista de Medicina, São Paulo, SP, Brasil; 6 Faculdade de Medicina da Universidade de São Paulo Laboratório de Investigação Médica (LIM-02) Departamento de Cirurgia São Paulo SP Brasil Departamento de Cirurgia, Laboratório de Investigação Médica (LIM-02), Faculdade de Medicina da Universidade de São Paulo, São Paulo, SP, Brasil; 7 Universidade de São Paulo Faculdade de Medicina São Paulo SP Brasil Faculdade de Medicina da Universidade de São Paulo, São Paulo, SP, Brasil; 8 Universidade Federal do Rio Grande do Norte Centro de Ciências da Saúde Natal RN Brasil Universidade Federal do Rio Grande do Norte, Centro de Ciências da Saúde, Programa de Pós-graduação em Ciências da Saúde, Natal, RN, Brasil

Dear Editors and Colleagues

We have examined with attention the comments of the letter to the Editor in regards to our recently published article (1), and want to thank its authors for their interest in our work.

After evaluating it, we would like to re-emphasize that have very different points of view, indeed!

Two aspects deserve to be highlighted:

1. We strongly believe that any new complication event which was added by this new technique, thus not being ever observed in traditional and conventional thyroidectomy, must not ever be considered as “minor complication”.
2. We positively do not consider that the technique presented by your group should be called “minimally invasive.” As a matter of fact, the TOETVA involves extensive soft tissue manipulation, as well as the need to use gas insufflation (which was long time ago abandoned, when the endoscopic thyroidectomies were initially described) and a significant increase ein operative time, compared to the traditional and conventional thyroidectomy

Last, but not least, there have been some serious complications, some even life-threatening, which have not been mentioned.

Therefore, looking from a bioethical approach, we encourage you to participate in a multi-institutional prospective peer-reviewed trial to offer a final and sound conclusion concerning the feasibility of TOETVA in Brazil. Until then, these procedures must be considered experimental, and its execution restricted to centers involved in this afore mentioned protocol.
